# The Role of Rab3a in Secretory Vesicle Docking Requires Association/Dissociation of Guanidine Phosphates and Munc18-1

**DOI:** 10.1371/journal.pone.0000616

**Published:** 2007-07-18

**Authors:** Jan R.T. van Weering, Ruud F. Toonen, Matthijs Verhage

**Affiliations:** Functional Genomics, Center for Neurogenomics and Cognitive Research, Vrije Universiteit Amsterdam, Amsterdam, The Netherlands; National Institutes of Health, United States of America

## Abstract

Rab3a is a small GTPase that binds selectively to secretory vesicles and switches between active, GTP-bound and inactive, GDP-bound conformations. In yeast, Rab and SM-genes interact genetically to promote vesicle targeting/fusion. We tested different Rab3a conformations and genetic interactions with the SM-gene *munc18-1* on the docking function of Rab3a in mammalian chromaffin cells. We expressed Rab3a mutants locked in the GTP- or GDP-bound form in wild-type and *munc18-1* null mutant cells and analyzed secretory vesicle distribution. We confirmed that wild-type Rab3a promotes vesicle docking in wild-type cells. Unexpectedly, both GTP- and GDP-locked Rab3a mutants did not promote docking. Furthermore, wild-type Rab3a did not promote docking in *munc18-1* null cells and GTP- and GDP-Rab3a both decreased the amount of docked vesicles. The results show that GTP- and GDP-locked conformations do not support a Munc18-1 dependent role of Rab3a in docking. This suggests that nucleotide cycling is required to support docking and that this action of Rab3a is upstream of Munc18-1.

## Introduction

Over 60 different Rab proteins are described in mammalian cells as key modulators of transport vesicles in the exocytotic and endocytotic pathway [1]. The subfamily of Rab3 proteins consists of four isoforms that are all expressed in secretory cells [2] and that bind specifically to synaptic and other secretory vesicles in the active, GTP-bound state [3], [4]. GTP-Rab3 isoforms interact with several proteins involved in vesicle secretion like Rabphilin-3a and RIM [5], [6] and dissociate from these proteins and the vesicle upon GTP hydrolysis [7], [8]. In the GDP-bound state, Rab3 isoforms are retrieved to the cytosol by GDP Dissociation Inhibitor to be able to associate again to secretory vesicles [9].

Rab3 isoforms have been implicated in several steps of regulated secretory pathways [10]. In general, both overexpression as well as null mutation of Rab3 isoforms appears to inhibit the final step: overexpression of Rab3 isoforms reduce secretion in PC-12 and chromaffin cells [11], [12], but null mutation of all four Rab3 genes also reduces secretion in cultured neurons, probably of a subpopulation of synaptic vesicles [13], [14]. However, this effect on the final secretion step may (in part) be explained by action of Rab3 in more upstream steps. In yeast and in endosome fusion, Rab proteins regulate vesicle docking in conjunction with Sec1/Munc18 proteins [15], [16] and in PC-12 cells, Rab3a overexpression increases docking while knock-down leads to fewer vesicles near the membrane [17], [18]. Even further upstream, rab3 appears to regulate synaptic vesicle targeting in *C. Elegans* [19] and mammalian nerve terminals [20].

To specifically address the cascade of events upstream of secretion, we examined, in analogy with yeast and endosomes, the actions of Rab3a in *munc18-1* null mutant cells. In addition to the wild-type Rab3a protein, also two mutants were expressed that are locked in the GTP and GDP bound form and that are expected to block Rab3a's actions at the target and donor side, respectively.

## Results and Discussion

### Rab3a cycling stimulates docking of large dense core vesicles (LDCVs)

We overexpressed Rab3a using Semliki Forest viral particles containing Rab3a and EGFP separated by an internal ribosomal entry site (IRES) in mouse chromaffin cells. Like endogenous Rab3a, all Rab3a constructs produced a punctate staining pattern, indicating that exogenous Rab3a is correctly translated and localized (Fig. S1A–D). The expression level did not differ significantly between Rab3a constructs (10 to 14 fold over endogenous Rab3a level) allowing direct comparison of their effects on the LDCV distribution (Fig. S1E).

**Figure 1:**
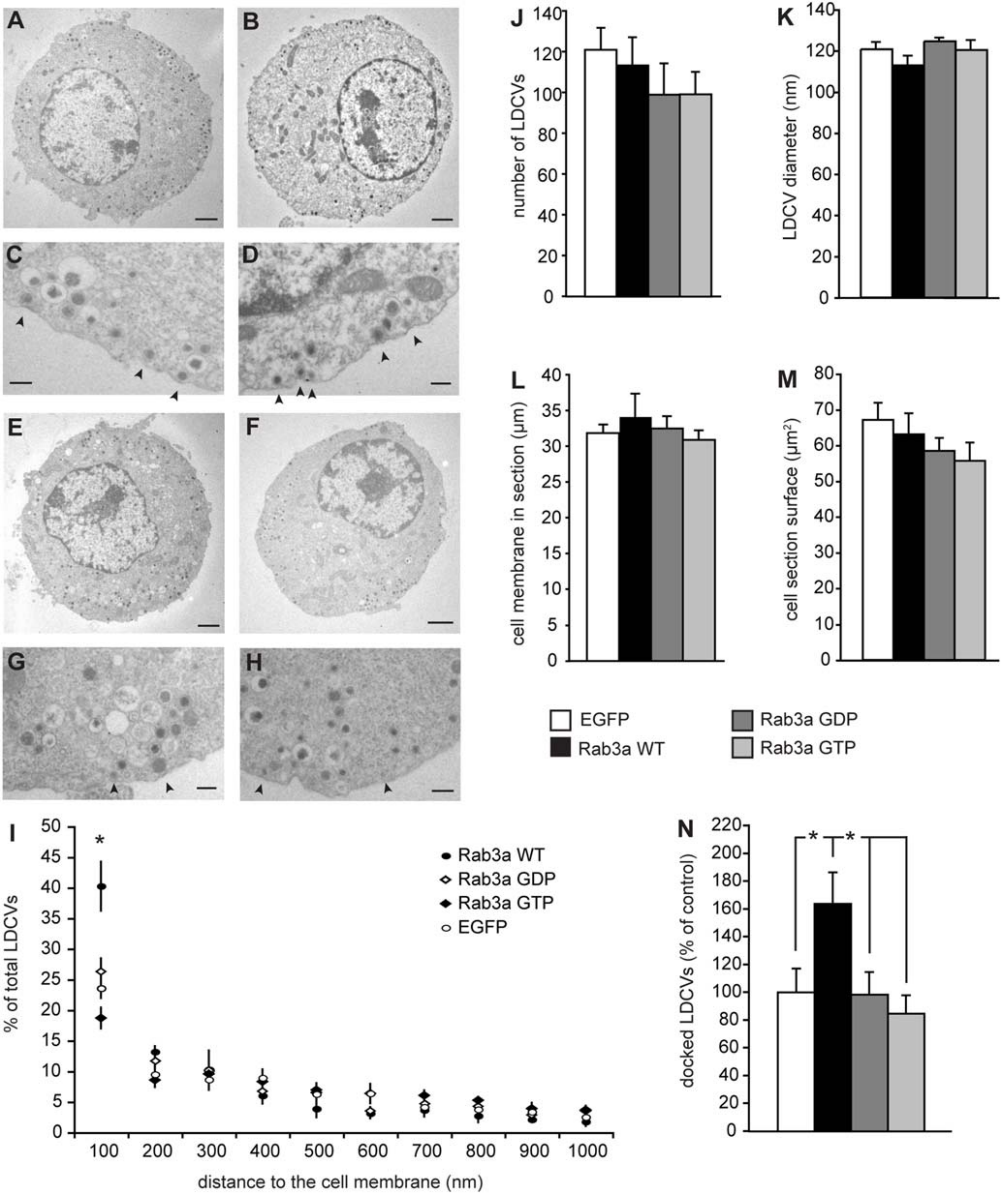
Switching between active and inactive Rab3a stimulates large dense core vesicle docking. Typical example micrographs of wild-type chromaffin cells expressing (A, C) IRES-EGFP control construct, (B, D) Rab3a wild-type-IRES-EGFP, (E, G) Rab3a GDP-IRES-EGFP and (F, H) Rab3a GTP-IRES-EGFP. (A, B, E, F) Examples of the total cell. Magnification 8,000×. Scale bar: 1 µm. (C, D, G, H) Detailed micrographs of the cell membrane region. Arrowheads indicate the morphological docked large dense core vesicles contacting the cell membrane. Magnification 20,000×. Scale bar: 200 nm. (I) Distribution profile of LDCVs in the cell section as percentage of the total number LDCVs present. Asterisk indicate significant difference between Rab3a wild-type and EGFP control as determined by T-Test (p<0.05). ANOVA on all groups p = 0.002. Other quantified parameters are shown in (J) the total number of LDCV present per cell section, (K) the average diameter of the LDCVs, (L) the length of the cell membrane in the cell section, (M) the surface area of the cell section in which the LDCV were quantified and (N) the size of the docked pool in the cell section as percentage of EGFP control. Asterisks indicate significant difference as tested by T-Test (p<0.05). ANOVA on all groups p = 0.023. All errorbars represent SEM.

Overexpression of wild-type Rab3a or the Rab3a mutants in wild-type chromaffin cells did not alter the cell morphology, the total amount of LDCVs or the vesicle size (Typical examples Fig. 1A, B, E, F; quantification in Fig. 1J, K, L, M). The general distribution profile of the LDCVs was not altered except for the region within 100 nm distance to the cell membrane (Fig. 1I). Wild-type Rab3a overexpression increased the amount of LDCVs in direct contact with the membrane (morphologically docked LDCVs: arrows in Fig. 1C, D; Fig. 1N), as observed before in a PC12 cell line [17]. Surprisingly, Rab3a locked in the GDP- or GTP-bound conformation did not affect the number of docked LDCVs, indicating that the active conformation is not sufficient to promote docking (arrows in Fig.1G, H; Fig 1N). Since all Rab3a proteins are expressed to similar levels (Fig. S1E), enhanced breakdown, misfolding or mistargeting of GTP- and GDP-Rab3a cannot explain the absence of effect on LDCV docking. Hence, it can be concluded that the ability to associate/dissociate GTP and GDP is essential for the function of Rab3a in docking.

### Rab3a effects on vesicle docking depend on Munc18-1

**Figure 2:**
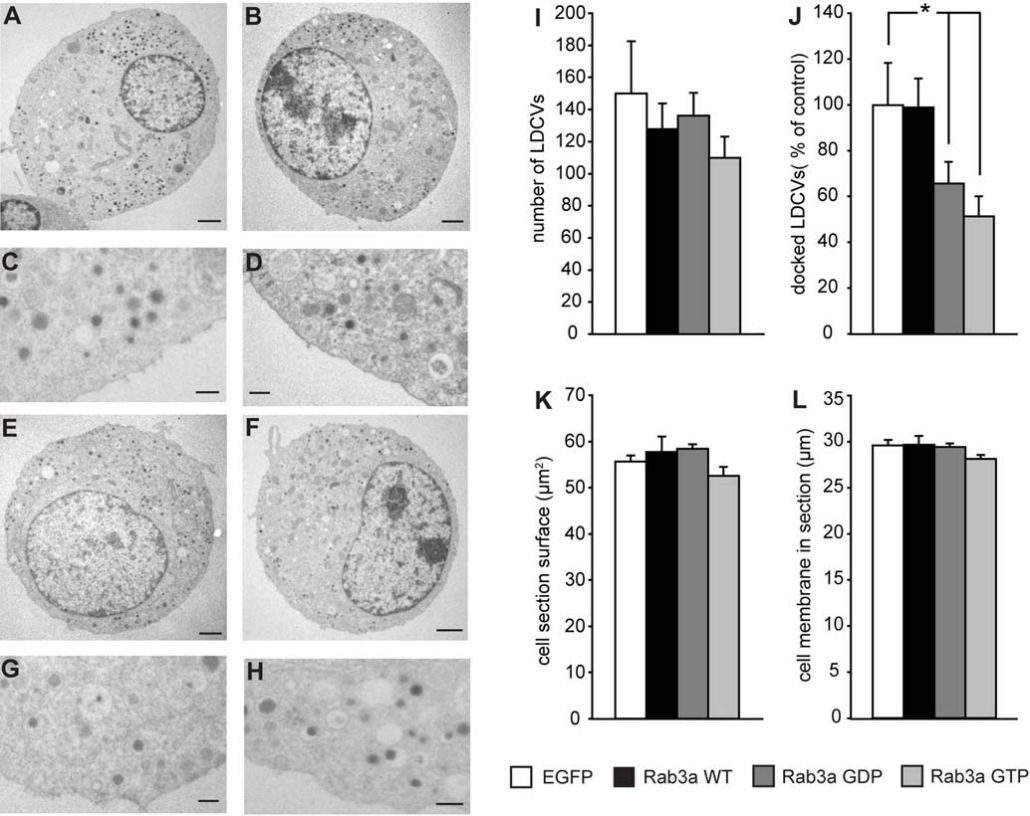
Rab3a depends on Munc18-1 to potentiate LDCV docking. Example micrographs of Munc18-1 knockout chromaffin cells expressing (A, C) the contol IRES-EGFP construct, (B, D) Rab3a wild-type-IRES-EGFP, (E, G) Rab3a GDP-IRES-EGFP and (F, H) Rab3a GTP-IRES-EGFP. (A, B, E, F) Examples of the total cell. Magnification 8,000×. Scale bar: 1 µm. (C, D, G, H) Detailed micrographs of the cell membrane region. Magnification 20,000x. Scale bar: 200 nm. Quantified parameters are shown in (I) the total number of LDCV present per cell section, (J) the amount of the docked LDCVs in the cell section as percentage of EGFP contol. Asterisk indicate significant difference as tested by T-Test (p<0.05); ANOVA on all groups p = 0.037. (K) The surface area of the cell section in which the LDCV were quantified. (L) The cell membrane length in the cell section. All errorbars represent SEM.

In other systems, Rab proteins regulate docking in conjunction with S/M proteins [15], [16]. Therefore, we tested the docking effect of Rab3a in *munc18-1* null mutant cells that are impaired in LDCV docking [21]. In contrast to wild-type chromaffin cells, Rab3a did not alter the number of docked LDCVs in the *munc18-1* null mutant cells (Fig 2 C, D; Fig. 2J). This indicates that Rab3a's action on LDCV docking depends on Munc18-1. Surprisingly, both GDP- and GTP-Rab3a reduced the number of docked LDCVs (Fig 2G, H; Fig 2J). The total number of LDCVs or the cell morphology of *munc18-1* null mutant chromaffin cells was not altered by either wild-type Rab3a or the two mutants (Typical examples Fig. 2A, B, E, F; Quantification Fig. 2I, K and L). This result suggests that locking Rab3a in GTP- or GDP-conformation reduces LDCV docking but this negative effect can be compensated in the wild-type cells by a Munc18-1 dependent process.

We conclude that switching between an active GTP-bound and inactive GDP-bound conformation of Rab3a promotes LDCV docking in a Munc18-1 dependent process. Switching between GDP- and GTP-bound conformations allows Rab3a to bind to several proteins and subsequently dissociate from them. In yeast, Rab protein Vps21 and S/M protein Vps45 interact via Vac1 to mediate vesicle fusion and Rab5 mediates homotypic fusion of endosomes via Rabenosyn-5 that associates with hVps45 [15], [16]. In LDCV secretion, Granuphilin/Slp4-a interacts with both Munc18-1 and Rab3a in pancreatic β-cells [22]. However, endogenous Granuphilin/Slp4-a is only detected in pancreas and pituitary suggesting a specific function in those tissues rather than a general role in vesicle secretion [23]. Our results suggest that both the association and the dissociation of a protein complex containing Rab3a and a functional homologue of Vac1, Rabenosyn-5 or Granuphilin/Slp4-a are important for LDCV docking.

## Materials and Methods

### Preparation of the Rab3a plasmids and Semliki Forest Virus production

Rab3a was cloned from mouse brain cDNA. Rab3a T36N (GDP-bound form) and Rab3a Q81L (GTP bound conformation) [24] were created by using QuickChange™ (Stratagene Cloning Systems, USA). All constructs were sequence verified. Rab3a constructs were cloned into pIRES2-EGFP vector (Clontech, USA) and Rab3a-IRES-EGFP was subsequently cloned into Semliki Forest Virus vector. Semliki Forest virus was produced according to [25].

### Cell culture

Adrenal chromaffin cells from E18 *munc18-1* null mutant mice and wild-type littermates were obtained according to [26]. The cells were infected with Semliki Forest virus at 2 DIV for 8 to 10 hours. The virus stocks are coded to perform the experiments blind.

### Immunocytochemistry

Cells were fixed in paraformaldehyde in PBS and stained with monoclonal Rab3a specific antibody (Cl42.2; Synaptic Systems, Germany) and secondary antibody goat-anti-mouse Alexa 543 (Molecular Probes, USA). Fluorescent signal was imaged on a Zeiss 510 microcope with a 63× objective using fixed laser settings and exposure time to allow quantative comparison. Average fluorescence intensity of EGFP and the Rab3 antibody staining were quantified using ImageJ (National Institute of Health, USA). All experiments were blinded for the type of virus used.

### Flat Embedding and Electron Microscopy

The location of infected cells on gridded coverslips (Bellco Glass Inc. USA) was photographed before fixing in glutaraldehyde in 0.1 M cacodylate buffer. The cells were prepared for electron microscopy like described in [26]. Infected cells were identified at low magnification in a JEOL 1010 electron microscope using the light microscope pictures and by the presence of virus-particles on the cell membrane of the cell. The distribution of LDCVs was examined at 20,000× magnification. Measurements were performed using analySIS (Soft Imaging Systems, Germany). LDCVs were recognized by their round dense core surrounded by a membrane and a diameter of approximately 100 nm. All experiments were performed blind for the type of virus used.

## Supporting Information

Figure S1 All Rab3a constructs are overexpressed approximately tenfold and immunostaining reveals a punctuated distribution. Normal chromaffin cells were infected with Semliki Forest viral particles containing Rab3a-IRES-EGFP sequence. Localization and expression level of Rab3a was determined by monoclonal Rab3a specific antibody Cl 42.2 and secondary antibody goat-anti-mouse Alexa 543. We used fixed laser settings for quantitative comparison between cells. Typical examples are shown of chromaffin cell expressing (A) the control IRES-EGFP construct, (B) Rab3a wild-type-IRES-EGFP, (C) Rab3a GDP-IRES-EGFP and (D) Rab3a GTP-IRES-EGFP. Scale bar: 2 µm. (E) Quantification of the average Rab3 signal in the cell. Asterisk indicate significant difference as tested by T-Test (p<0.05). ANOVA on all groups p = 1.02×10-7. Error bars represent SEM.
(1.77 MB TIF)
