# Supplementary figures and images for: The Role of Rab3a in Secretory Vesicle Docking Requires Association/Dissociation of Guanidine Phosphates and Munc18-1
Source: PLoS One. 2007 Jul 18;2(7):e616. doi: 10.1371/journal.pone.0000616 (PMC1910611; doi:10.1371/journal.pone.0000616)

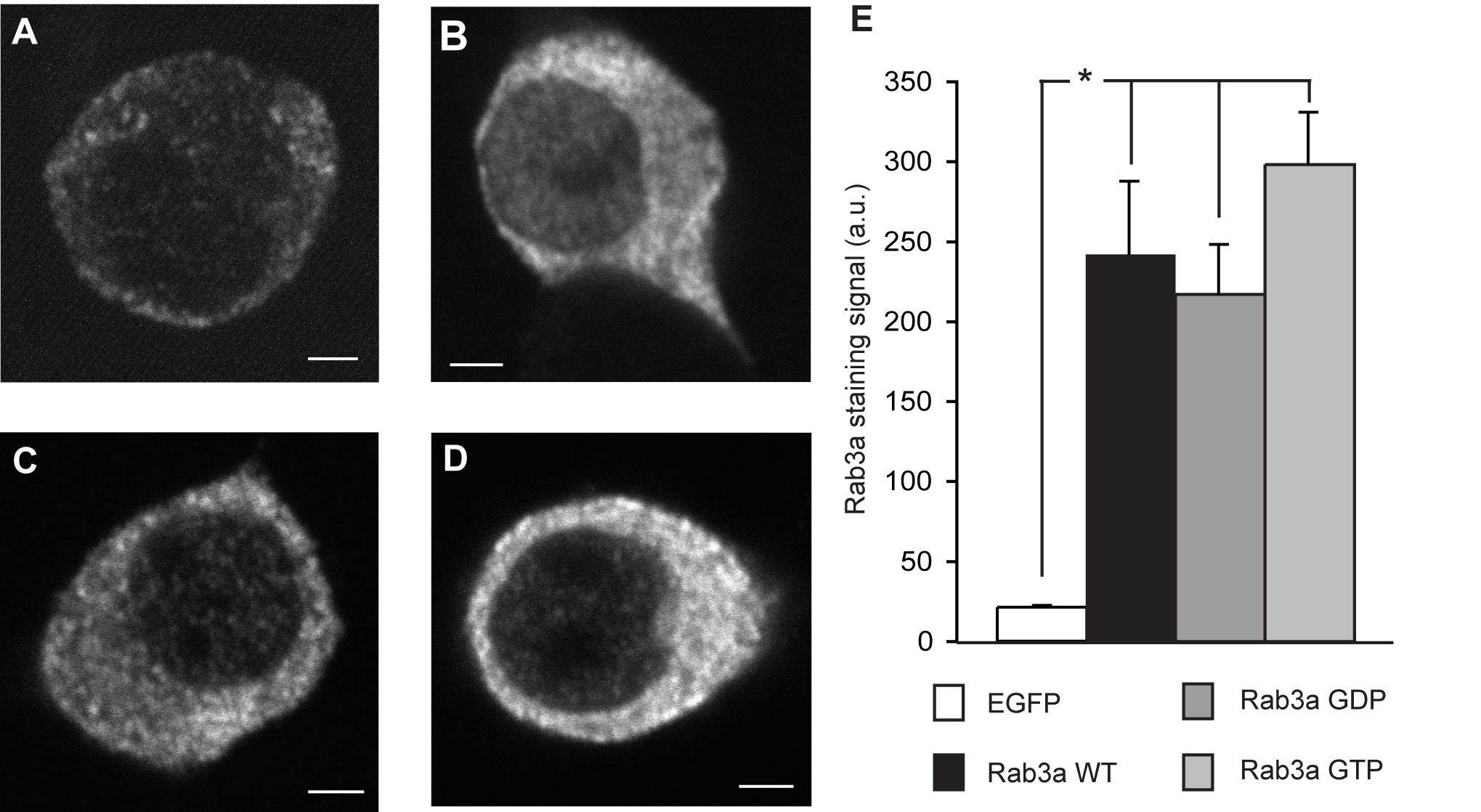

Supplement: Figure S1 — All Rab3a constructs are overexpressed approximately tenfold and immunostaining reveals a punctuated distribution. Normal chromaffin cells were infected with Semliki Forest viral particles containing Rab3a-IRES-EGFP sequence. Localization and expression level of Rab3a was determined by monoclonal Rab3a specific antibody Cl 42.2 and secondary antibody goat-anti-mouse Alexa 543. We used fixed laser settings for quantitative comparison between cells. Typical examples are shown of chromaffin cell expressing (A) the control IRES-EGFP construct, (B) Rab3a wild-type-IRES-EGFP, (C) Rab3a GDP-IRES-EGFP and (D) Rab3a GTP-IRES-EGFP. Scale bar: 2 µm. (E) Quantification of the average Rab3 signal in the cell. Asterisk indicate significant difference as tested by T-Test (p<0.05). ANOVA on all groups p = 1.02×10^-7^. Error bars represent SEM. (1.77 MB TIF) [file pone.0000616.s001.tif]
